# Additional Observations upon Veratrum Viride

**Published:** 1857-10

**Authors:** V. H. Taliaferro

**Affiliations:** Atlanta, Geo.


### ARTICLE II.

*Additional Observations upon Veratrum Viride.* By V. H. Taliaferro, M. D., Atlanta, Geo.

Some time since I prepared an article for the Atlanta Medical and Surgical Journal, upon Veratrum Viride, which was published in the March No., 1857, since which time I have continued my observations with veratrum, being in the habit of almost daily prescribing it, and my conclusion most irresistably is, that it is a remedy of such important therapeutic properties, as to demand from the profession a fair and unprejudiced investigation.

The profession, however, as in this case, are too much accustomed, in the investigation of new remedies, to laud their virtues alone, and leave unnoticed the peculiar circumstances under which their administration would be improper. When the therapeutic application of remedies to disease are fully appreciated, we are enabled with comparatively little difficulty, to point out conditions contra-indicating their use; as in the case of opium, mercury, arsenic, antimony, quinine, &c., and in fact, all powerful remedial agents, which are well known to be frequently contra-indicated by circumstances connected with the diseases to which they are applicable. And thus it is with the remedy under consideration.

We are well satisfied that the diseases to which veratrum is particularly applicable, are those of an inflammatory character; and this peculiar applicability is plain, when we consider its influence upon the circulatory system, controlling excessive action of the heart and arteries, which is an invariable consequence of high grades of inflammatory action.

It is upon this principle, we contend, that veratrum often answers the purpose of the lancet in inflammatory affections. We have found it more effectual, prompt, and decided in the treatment of *pneumonia* than in any other disease, whether sthenic or asthenic in its character. Its sedative action upon the circulation, its relaxing and expectorant properties render it peculiarly adapted to the treatment of this disease. But, notwithstanding this peculiar applicability, and the great advantage to be derived from its use in the treatment of pneumonia, we have been led to the conclusion, that in certain stages of the disease, it should be used with much circumspection, viz: in hepatization of the lungs. If either lobe of the lungs become hepatized, the other, perchance, inflamed, the circulation of the blood *must*, of necessity, become much accelerated, as *must* also the inspiration of oxygen, to properly arterialize the circulating fluid, in consequence of the diminished amount of blood exposed to the action of atmospheric air at each inspiration. It must be evident, then, that if under these circumstances, we compel the heart to beat its accustomed number of pulsations, for any given time, the blood must become imperfectly oxygenized.

Upon these considerations, then, we are forced to the conclusion, that veratrum viride, in hepatization of the lungs, should be administered with much care and circumspection, attention being continually directed to the number of pulsations of the heart per minute during its use.

We would not wish to be understood as condemning entirely the use of veratrum in hepatization of the lungs; on the other hand, we would advise it as important, but would recommend a more particular attention to its effects upon the circulation than is requisite under more favorable circumstances for its administration. That the heart may be made to pulsate too slowly for the proper arterialization of the blood is evident; if so, then the above conclusion is natural and reasonable.

As the capacity for the reception of venous blood in the lungs is lessened, in the same proportion will the heart's action become accelerated, in order to supply the demand of the system for oxygen. If, then, this accelerated action is the effect of diminished capacity of the lungs, is it not plain that if it is injudiciously interfered with, without at the same time enlarging the lung capacity, that we deprive the blood of its natural supply of oxygen? Every one has noticed the rapid respiration of the female whose chest is tightly laced, and if the pulse be then examined, it will be found to be much accelerated, corresponding to the rapidity of respiration, and it is evident that it is the result of the lungs' diminished capacity. Suppose we administer to such an individual veratrum viride, until its specific action is obtained upon the circulation, is it not plain that unless we then relieve the lungs from its compression, that we very soon would have the system diffused with imperfectly arterialized blood and its sequences. So would follow the same result in hepatization of the lungs.

In typhoid fever the virtues of veratrum are not so fully and effectually displayed, but may be used advantageously as an adjuvant to other and more supporting treatment. We think much discrimination should be exercised in carrying it to the extent of nausea and great muscular relaxation, in this disease, from the fact that nervous tone and energy, under such circumstances, would be materially lessened, while one of the great indications to be fulfilled in the treatment, is the maintenance of nervous energy by giving, as far as possible, tone and strength to the whole system.

Our experience with veratrum has taught us that under ordinary circumstances, in the treatment of any disease, it is unnecessary to carry its effects to the extent of great muscular and nervous prostration; but more especially in typhoid fever, we believe discrimination should be exercised in producing its specific action upon the circulation, for fear of nausea and muscular relaxation.

We consider veratrum an important addition to our list of therapeutic agents, and doubt not that science and experience will 'ere long give it that exalted position which its intrinsic merits so justly demand; but we are forced to the conclusion that Dr. Norwood has materially lessened the interest the profession would have evinced in its investigation, by endeavoring to leave them under the impression that unless *his* preparation [" *Our Tinct.*"] was purchased at an extravagant price, it must of necessity be spurious. Dr. N. has either become a monomaniac, or else allowed pecuniary considerations to have led him from the track of an honorable and scientific profession, by thus sacrificing all professional pride. It is of frequent occurrence for unprincipled empirics to lead astray the populace by their nefarious pretensions, but it is of somewhat rare occurrence for an M. D. to attempt the practice of empiricism and quackery upon the *medical profession* with the boldness with which Dr. N. proposes his infallibles for scald-head, convulsions, &c. Pity for Dr. Norwood that he had not forsaken his pen before publishing to the world that he was soon to bless the profession with a cure for scald-head, &c., besides endeavoring to lead them into the erroneous impression that they could not obtain pure, an indigenous plant, growing over many States of the Union, unless submitted to his inspection, or its tincture, unless prepared under his supervision.

